# Is Language Production Planning Emergent From Action Planning? A Preliminary Investigation

**DOI:** 10.3389/fpsyg.2020.01193

**Published:** 2020-06-05

**Authors:** Mark J. Koranda, Federica Bulgarelli, Daniel J. Weiss, Maryellen C. MacDonald

**Affiliations:** ^1^Department of Psychology, University of Wisconsin-Madison, Madison, WI, United States; ^2^Department of Psychology, Pennsylvania State University, State College, PA, United States; ^3^Department of Psychology and Neuroscience, Duke University, Durham, NC, United States

**Keywords:** language emergence, language production, action planning, syntactic priming, hysteresis, domain general processing

## Abstract

The nature of syntactic planning for language production may reflect language-specific processes, but an alternative is that syntactic planning is an example of more domain-general action planning processes. If so, language and non-linguistic action planning should have identifiable commonalities, consistent with an underlying shared system. Action and language research have had little contact, however, and such comparisons are therefore lacking. Here, we address this gap by taking advantage of a striking similarity between two phenomena in language and action production. One is known as syntactic priming—the tendency to re-use a recently produced sentence structure—and the second is hysteresis—the tendency to re-use a previously executed abstract action plan, such as a limb movement. We examined syntactic priming/hysteresis in parallel language and action tasks intermixed in a single experimental session. Our goals were to establish the feasibility of investigating language and action planning within the same participants and to inform debates on the language-specific vs. domain-general nature of planning systems. In both action and language tasks, target trials afforded two alternative orders of subcomponents in the participant’s response: in the language task, a picture could be described with two different word orders, and in the action task, locations on a touch screen could be touched in two different orders. Prime trials preceding the target trial promoted one of two plans in the respective domain. Manipulations yielded higher rates of primed behavior in both tasks. In an exploratory cross-domain analysis, there was some evidence for stronger priming effects in some combinations of action and language priming conditions than others. These results establish a method for investigating the degree to which language planning is part of a domain-general action planning system.

## Introduction

A key component of action planning is implicit decision making (Wolpert and Landy, 2012), where actors settle on choices among viable options to meet task goals. Choices can include using the left vs. the right hand for some action, or reaching for a spoon first and then a fork vs. the other way around. Language use, which Lashley (1951) discussed as a form of action, requires similar implicit decisions among alternative language forms to convey the producer’s message. For example, speakers make word choices, such as describing a piece of furniture as either a *sofa* or a *couch*. Correspondingly, they also have options for different hierarchical sentence plans or syntactic structures, which generally result in different serial orders of words, as in *Maya gave the old sofa to her brother* vs. *Maya gave her brother the old sofa.* A number of researchers have followed Lashley in pointing to potential parallels between action and language and considered the degree to which properties of language can be seen as emergent from more general action systems (Steedman, 2002; Arbib, 2006; Garrod and Pickering, 2009; Fitch and Martins, 2014; Vicari and Adenzato, 2014; Casado et al., 2018). Yet, action and language also clearly differ in myriad ways, and these differences can make it difficult to evaluate any claims of relationships between the two systems. Here, we report preliminary steps in investigating this relationship via tasks that are designed to have key components in common. Our focus is not at comparatively low levels for which it would not be surprising to find commonalities, such as motor control of the vocal tract for speech and of the hands for grasping (Sevald and Dell, 1994). Instead, we focus on higher levels of language and action production, the role of prior experience on serial ordering in producing sentences and actions. Because syntactic processes are often claimed to be language-specific, investigating potential commonalities between syntactic planning and non-linguistic action planning has good potential to advance the dialog between language and action research.

In both the action and language domains, the probability of making alternative choices is known to vary as a function of prior action. For example, in motor reaching tasks, hand selection is often influenced by which hand the actor used in recently performed actions (Rostoft et al., 2002; Weiss and Wark, 2009; Valyear and Frey, 2014; Valyear et al., 2019). These behaviors are examples of *hysteresis*, a term that broadly refers to how physical systems are impacted by their prior history. Relevant to our study, hysteresis in motor control has often been described in terms of asymmetries in motor behaviors on the basis of prior executions. Notably, in sequential choice behaviors, repeating action plans may be more cognitively economical, as it is thought to be easier to select a previously executed plan rather than creating a new one from scratch (Rosenbaum et al., 2007). For instance, when actors transport an object from one location to another, they are more likely to re-deploy the previous grasp when returning the object to its initial location rather than selecting a locally optimal grasp (Cohen and Rosenbaum, 2004). This tendency can be accounted for by models such as the posture-based motion planning theory, which suggests that goal postures involve the selection of a stored posture that is subsequently modified for the execution of a new movement (see Rosenbaum et al., 2006). Such cognitive accounts of plan reuse have recently been termed the computational efficiency model of action hysteresis (Valyear et al., 2019), as the selection of recently executed plans correlate with reduced response times.

In language, speakers repeat recently used words (Clark and Wasow, 1998), sentence structures (Bock, 1986), and other aspects of language at higher than chance rates. Similar to the action domain, discussions of computational efficiency also are important in accounts of these behaviors, which are typically described as *priming* or *persistence* effects (Bock, 1986; Ferreira and Bock, 2006). For example, speakers reuse abstract sentence (syntactic) plans even when there is no overlap in topic or words from one sentence to another, and there is some evidence that such reuse improves speaking fluency (Corley and Scheepers, 2002). The phenomenon was first described by Weiner and Labov (1983), who studied the sentence structures produced by speakers during natural conversations. They found that a strong predictor of a speaker producing a rare sentence structure (a passive sentence such as *The book was found*) was whether that person had previously produced a passive sentence earlier in the conversation. Following these early naturalistic observations, *syntactic priming* or *structural persistence* effects have been abundantly documented in laboratory studies, typically in designs in which participants repeat or read aloud one or more sentences containing a particular sentence structure, which serves to “prime” that sentence structure, followed by presentation of an unrelated picture that participants must describe. The dependent measure is the extent to which participants’ picture descriptions use the same sentence structure and serial order of phrases as in the prime sentence(s) (Bock, 1986; McDonald et al., 1993). Structure priming effects in language production have been shown to be subtle but reliable across different sentence types and task variations, in both children and adults, and in a number of different languages (Mahowald et al., 2016).

Together, this work in action and language domains suggests that in both cases, actors must make implicit choices, including choices that affect serial ordering of subcomponents of the action. Moreover, in both cases, serial ordering choices are known to be influenced by serial ordering of actions/language executed in the recent past. On this view, there could be benefit to investigating the degree to which these language and action behaviors as examples of a broader tendency in both systems toward efficiency-based plan reuse (MacDonald, 2013). Despite potential parallels, however, the fields of action behavior and language production have largely been studied separately, and often with different theoretical accounts for the origin of these reuse effects. This lack of integration across fields is unfortunate, because similarities in plan reuse in the two domains could be a route for theoretical development in each field, spurred by consideration of results from the other field. Better integration across fields also can promote broader theoretical consideration of the extent to which language production processes can be seen as emergent from more domain general sequencing processes. Here, we take initial steps to bridge these two areas by designing an action and a language task with similar serial ordering components, creating an environment in which we can more formally investigate potential parallels between implicit serial ordering choices in each domain as a function of prior action.

We developed a language production task and an action task with parallel task structure, designed to allow trials from both tasks to be interleaved within a single experiment. In both tasks, a target trial required sequencing of several subcomponents: hand movements in the action task in order to touch target locations on a computer screen, and phrases to be spoken in the language task in order to describe a picture presented on the computer screen. In both tasks, either order of subcomponents allowed participants to complete the trial successfully. In order to test plan reuse on subcomponent ordering, target stimuli were preceded by two “prime” stimuli in which task subcomponents had to be executed in a specified order—a particular navigation path in the action task and a particular syntactic order in the language task. The dependent measure was the order of subcomponents produced in each type of target trial, as a function of the ordering that was fixed by the preceding prime trails. If plan reuse (hysteresis and syntactic priming) operates in both domains, then on target trials, participants should tend to produce the subcomponent orders (hand movements or phrases) that match the orders that were produced in the immediately preceding prime trials.

## Materials and Methods

### Participants

Participants were 98 Native English speakers; 42 from the University of Wisconsin-Madison (female = 27, age not collected) and 56 from Pennsylvania State University (female = 50, age *M* = 18.5, *SD* = 0.99). Participants completed the experiment for course credit or pay and their data were used for subsequent analyses. This study was approved by the universities’ IRB boards, and all participants provided written informed consent.

An additional 33 participants were excluded because of failure to follow instructions (24), because participants had indicated an awareness of the priming manipulation in either the language or action task (4), non-native speaker status (2), or technical difficulties (3). All participants were right-handed.

### Materials

Three types of stimuli were developed for both the action task and the language task. In both tasks, target stimuli afforded two alternative responses that differed in the order of their subcomponents: ordering of hand movements in the action task, ordering of phrases in the language task. Prime stimuli required an ordering of these subcomponents via stimuli that afforded only one response option. Filler trials were placed in between prime-target sequences in order to minimize participants’ detection of prime-target relationships; filler stimuli were designed not to prime either of the responses available on target trials.

#### Action Stimuli

**FIGURE 1:**
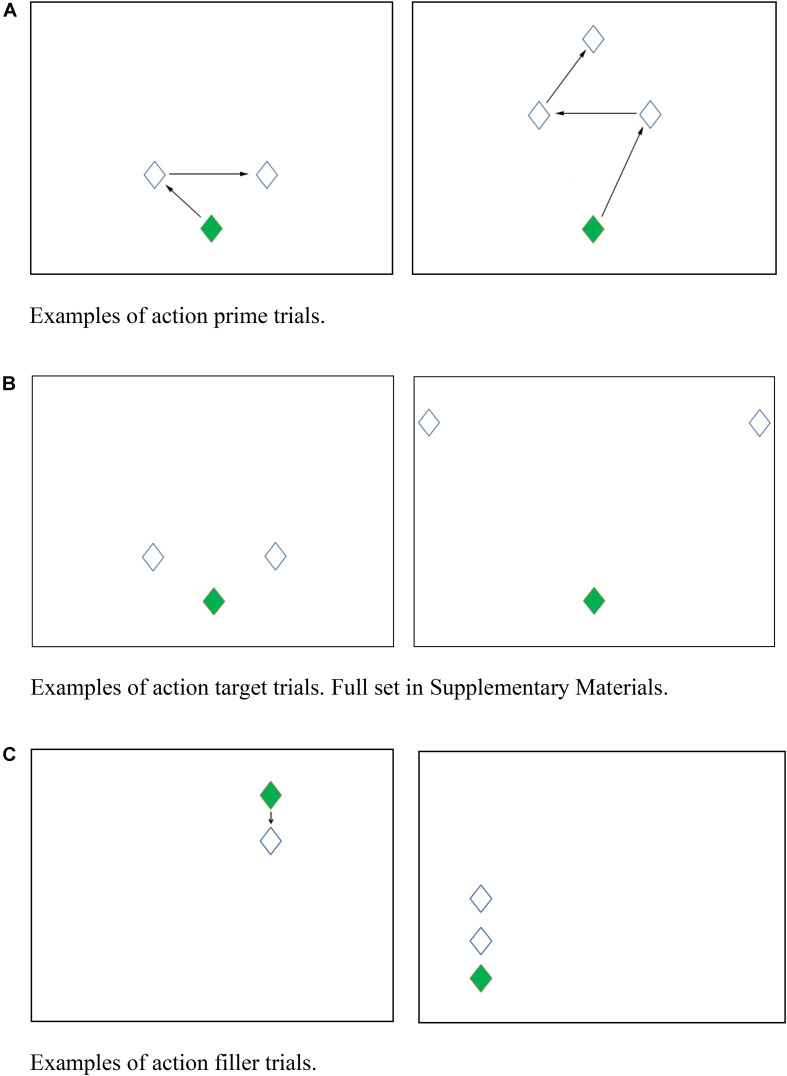
**(A)** Examples of action prime trials. **(B)** Examples of action target trials. Full set in Supplementary Material. **(C)** Examples of action filler trials.

All action stimuli were arrays of diamonds on a touch screen indicating locations where participants should touch. Each display had one green diamond designated as Start (the first to be touched in a trial) and one or more white diamonds (80 × 100 pixels, see Figure 1). For Prime trials (*n* = 24), the Start diamond was centered near the bottom of the screen, and two white diamonds were arranged symmetrically above and to the left and right of the green Start diamond. Arrows were placed between the diamonds to indicate the sequence in which the participants should touch the diamonds on screen (see Figure 1A).

Target trials (*n* = 12) were similar in layout to the prime trials but did not contain the arrows indicating the sequence in which the participant should touch the diamonds on screen, and thus they allowed an action sequence of touching the left diamond immediately after the Start diamond and then touching the right diamond (left-first order) or the opposite sequence (right-first order). For both Prime and Target trials, the exact screen position and the distance between the Start and white diamonds varied, but the two white diamonds above and to the left and right of the Start diamond were always equidistant from the Start diamond. A subset of the target trials (*n* = 4) also had an additional white diamond above the left and right diamonds which were centered above the green start diamond (see Figure 1B for examples). Across the prime and target trials, there was no exact repetition of screen positions for Start or white diamonds.

In addition to the Prime and Target trials, there were 36 filler trials, in which diamonds were arranged in a vertical line, varying in horizontal position, presence of arrows, distance between diamonds, and number of diamonds (see Figure 1C). The filler items all contained vertical arrays of diamonds, so that there was no priming of leftward or rightward hand movements in the filler trials.

#### Language Stimuli

Language stimuli also included prime, target, and filler trials. Stimuli for these trial types included printed sentences onscreen to be read aloud and pictures to be described.

**FIGURE 2:**
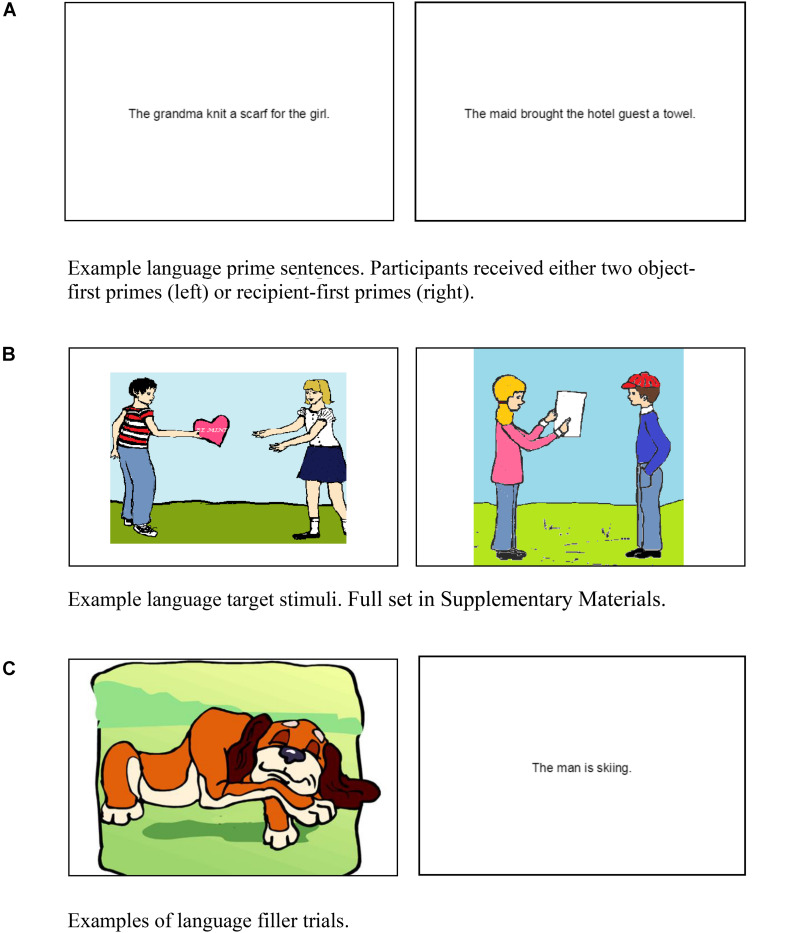
**(A)** Example language prime sentences. Participants received either two object-first primes (left) or recipient-first primes (right). **(B)** Example language target stimuli. Full set in Supplementary Material. **(C)** Examples of language filler trials.

Prime trials (*n* = 24) consisted only of sentences, which were presented centered on the computer screen. The sentences contained one of two word orders: an object first order such as: *The maid brought a towel to the hotel guest*, in which the object (towel) precedes the recipient (hotel guest), or a recipient-first order: *The maid brought the hotel guest a towel*, in which the recipient (hotel guest) precedes the object (towel). Object- and recipient-first structures are synonymous with Prepositional and Double Object Dative constructions, respectively. Examples are shown in Figure 2A.

Target trials (*n* = 12) consisted of pictures depicting an event in which one human transferred an inanimate object to another human or demonstrated something about an object to another human. For example, the left-hand picture in Figure 2B can be described with an object-first order, *The boy is giving a valentine to the girl*, or a recipient-first order, *The boy is giving the girl a valentine.* See the Supplementary Material for full set of target pictures. Some pictures were edited versions of ones given to the authors by Kay Bock, and others were developed with clipart. Because speakers can describe pictures in many ways beyond the language forms of interest (e.g., *Two kids are looking at something*, which has no mention of the recipient and vague mention of the object), the pictures were pilot tested and selected to be those that best elicited descriptions that consistently included mention of both humans and the inanimate object but no other detail (e.g., features of the background).

Language filler trials (*n* = 36) were a mix of sentence and picture items. Sentence fillers described simple intransitive events with a single human or animal doing an action with no object or recipient (e.g., *The man is skiing*). Picture fillers also depicted intransitive actions such as sleeping, stretching, running with one human/animal actor and no objects (see Figure 2C). For these trials, there was no option of alternate object-first or recipient-first ordering because there was neither an object nor a recipient in the picture or sentence.

### Procedure

Participants were tested at the Pennsylvania State University and the University of Wisconsin-Madison, using an identical Dell 23″ touch screen monitor with 1920 × 1080 resolution. The same Eprime 2.0 scripts and instructions were used at both sites, and the only difference was that participants’ spoken responses were recorded through the E-Prime 2.0 software in Wisconsin, whereas they were recorded separately using a Marantz© recorder in Pennsylvania.

Participants sat in front of the touchscreen and a microphone. They were informed they would see a mixture of different kinds of trials during the experiment, and that each trial would display either a sentence, a picture, or an array of diamonds. Participants were instructed to read sentences aloud and to describe pictures with a single sentence. For diamond arrays, participants were instructed to first touch the green Start diamond, then touch all remaining diamonds. If arrows were present, they were to touch the diamonds in the order indicated, otherwise they could touch the white diamonds in any order.

Following instructions, four practice trials were presented to familiarize participants with each trial type. These trials were identical in format to filler trials, in that they did not afford any sequencing options. They contained two touch-screen action trials (one with and one without arrows) and two language trials (one sentence and one picture). During the practice trials, the experimenter gave explicit feedback if participants did not follow instructions, or if verbal descriptions were missing elements or contained excessively elaborate descriptions (e.g., more than one superfluous element mentioned, such as including the color of items in the picture or describing the background).

**FIGURE 3:**
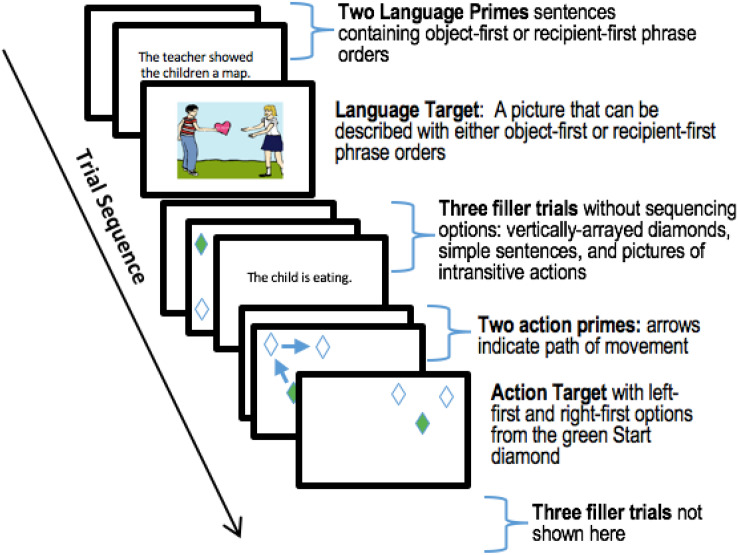
Trial sequence. Participants saw a series of interleaved, fully randomized action and language targets, with five trials between each target trial. A prime-target-filler sequence began with two prime trials, either two action or two language primes. A target of the same domain (action or language) followed the two prime trials. Three filler trials, which afforded no sequencing options, followed the target. Each group of three fillers contained at least one language and one action filler trial. In this figure, screen displays are grouped to illustrate the trial sequence, but each trial in the experiment proceeded immediately following the end of the previous trial.

The experiment comprised 144 trials: 48 primes trials, 24 target trials, and 72 fillers. The order of presentation for trials was as follows: two prime trials from a single domain (either both action prime trials or both language primes) were immediately followed by a target trial from the same domain as the preceding primes. Presenting two instead of one prime allowed us to minimize potential noise associated with the demands of task-switching amidst randomly interleaved action and language trials. Each target trial was followed by three randomly sampled filler trials. Each triplet of fillers contained at least one action filler trial and one language filler. Action and language prime-target sequences were randomly interleaved through the experiment, with 12 prime-target sets for each domain. An example is shown in Figure 3.

Participants were instructed to advance language trials (sentence or picture) by touching the screen once they finished speaking. Action trials automatically advanced after the participant touched the screen as many times as there were diamonds present. The experimenter sat next to the participant and ensured these instructions were followed.

Similar to the motivation for using two primes in a row, we sought to maximize likelihood of detecting an effect by implementing prime conditions between subjects. For the language trials, a participant received either object-first only or recipient-first only language primes. For the action trials, a participant received either left-first only or right-first only action primes. All combinations of prime types resulted in four lists: object-first and left-first primes, object-first and right-first, recipient-first and left-first, recipient-first and right-first. Participants received one of two random presentation orders.

Following the end of the experiment, participants were interviewed about what they had noticed about the tasks and what they thought that the task was about. Only about 3% of participants reported noticing the sequencing options in one or both types of trials. Those participants were eliminated from all analyses (as noted above in the “Participants” section).

### Data Coding

#### Screen Touches in the Action Task

Action target trials had two possible outcomes of interest, namely, whether the left or right diamond was touched first. Touches to the screen counted as touches to a diamond if they were within 45 pixels of the center of the nearest diamond. Valid trials were defined as having the same number of screen touches as diamonds on the screen, a first touch on the green Start diamond, and one of the diamonds to either side of the start diamond. A total of 65 trials (5.51%) were excluded by failing to meet one or more of these criteria. For all remaining trials, responses were coded as either leftward or rightward movement from start, i.e., whether the left or right diamond was touched immediately after touching start.

#### Utterances in the Language Task

A valid picture description response for language target trials required mention of both humans in the picture and the inanimate object being transferred in the scene, as well as using a verb for which both recipient-first and object-first word orders were possible (Bock, 1986). For example, *The waiter handed the woman a plate* was coded as a valid response, whereas *The woman received a plate of food* was invalid because the verb did not permit both word orders and because the waiter was not mentioned. A total of 372 language trials were excluded (31.60%; exclusions were equally frequent in each condition). This rate of exclusions is comparable to rates in other syntactic priming studies using picture description and reflects the fact that participants are not explicitly instructed to produce a particular kind of sentence (Bock, 1986).

Valid trials were coded as having the sentence structure of either object-first (e.g., *The nurse gave the cup to the boy*) or recipient first (*The nurse gave the boy the cup*).

## Results

### Overall Response Choices

Before we report effects of primes on target responses, we first report the overall rates of alternative responses on target trials in Table 1. As can be seen in the table, there was a strong preference for left-first responses over right-first responses in the action domain. This pattern was also found in pilot data without the language trials interleaved. The current study was not designed to investigate other dimensions influencing action biases, but one possible factor in the left-first bias is that when using the right hand to touch the screen (as all participants were required to do) a movement leftward toward the body may be easier than a rightward movement away from the body. Another reason for an overall left-first response bias may be that reading English text constrains eye fixations to be left-to-right ordered, potentially priming leftward eye fixations in the action task.

**TABLE 1:** Proportion of subcomponent ordering in responses by domain.

Response domain	Subcomponent order	*M*	*SD*
Action	Right-first	0.40	0.38
	Left-first	0.60	0.38
Language	Recipient-first	0.49	0.27
	Object-first	0.51	0.27

*Values are computed from by-subject means over all valid responses to target trials.*

In the language domain, participants did not exhibit a preference for either type of response. The overall language results are generally consistent with prior studies concerning rates of object-first and recipient-first sentences produced in language production tasks, in which a fairly even distribution of choices is found or a slight bias toward object-first structures, which varies with properties of the stimuli used to prompt language production and reflects the fact that implicit choices of alternative forms vary along multiple dimensions (Bock and Irwin, 1980; Bock, 1986; Bock and Loebell, 1990). For example, the visual scenes for eliciting the two sentence types of interest here typically depict demonstration of an object or transfer of possession of an object, as in Figure 2B. As this figure illustrates, the direct object is shown in between the agent and the recipient. The close proximity of the agent (which is mentioned before either object or recipient) and the object in the visual scene may promote object first descriptions. More generally, however, the effect of visual organization on sentence structure appears to be relatively minor (Bock et al., 2003).

### Priming Effects on Target Responses

Here, we analyze the rate of prime-congruent responses, so that effects in each domain can be described with parallel terminology. For example, a left-first response to an action target trial was coded as prime-congruent when it was preceded by left-first prime trials, and a left-first target response was coded as prime-incongruent when that target was preceded by right-first primes.

Linear mixed effects models (Judd et al., 2012) were used for all analyses in order to predict participants’ behavior in each task. Each target trial was coded as ‘1’ if the behavior was congruent with the prime or ‘0’ if the behavior was incongruent with the prime. All models contained maximal random-effects structures (by-item and by-subject random intercept and random slopes for all predictor variables), unless a model failed to converge. In those cases, planned steps were taken to achieve the most maximal model that could converge according to Barr et al. (2013).

#### Action Trials

**FIGURE 4:**
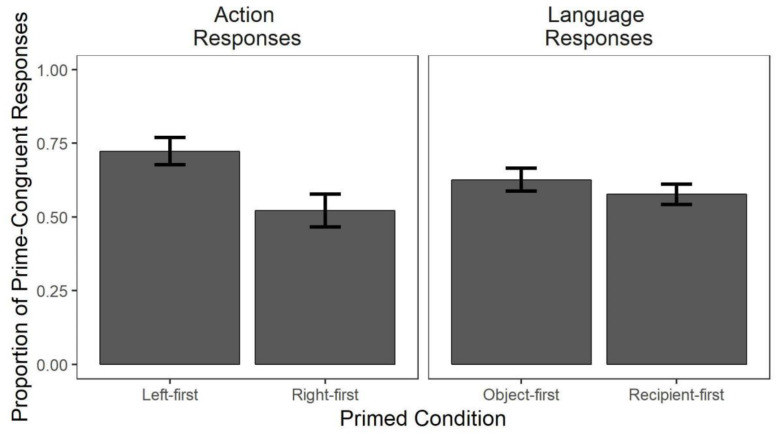
Proportion of target trials in which participants produced prime-congruent responses. For action targets, bars reflect the proportion of screen touches that match the primed direction from the Start diamond (left-first/right-first, totaling to 1). For language targets, bars reflect the proportion of utterances (object-first/recipient-first, totaling to 1) that matched the structure of the primed object/recipient ordering. Means and standard errors are calculated over each participant’s mean score. Overall, prime order predicts rates of prime-congruent vs. prime-incongruent responses in both action and language trials, and for action trials the size of priming is greater for left-first primes.

Rates of prime-congruent responses in the action domain are shown in the left panel of Figure 4. Analyses revealed a main effect of priming, meaning that on target trials, participants produced the prime-congruent responses reliably more often than prime-incongruent responses (*z* = 3.13, *p* < 0.05). Priming effectiveness was greater for left-first plans (*z* = −2.42, *p* < 0.05). That is, while both primes increased prime-congruent responses over prime-incongruent responses, the proportion of increase was significantly higher with left-first priming. Priming was also predicted by trial number (*z* = 4.06, *p* < 0.05), such that the degree to which the primed order was produced on target trials increased through the course of the experiment, potentially reflecting cumulative effects of priming; recall that prime type was manipulated between subjects, such that one prime (e.g., left-first) was used in all trials in a domain.

#### Language Trials

Rates of prime-congruent responses in the language domain are shown in the right panel of Figure 4. As in the action condition, language primes significantly predicted word order in picture descriptions on target trials, such that participants used the primed word order more than the prime-incongruent one (*z* = 3.70, *p* < 0.05). object-first and recipient-first primes did not differ in their priming effectiveness (*p* > 0.05). There was also no effect of trial number, meaning that rates of prime-congruent responses did not change through the course of the experiment.

A clear pattern in the data in the right panel of Figure 4 is that the serial ordering of task subcomponents on target trials was influenced by the prime trials in both the action and language domains. These results suggest that it is possible to design action and language tasks with broadly parallel structures to examine plan reuse effects in both domains, in the same participants and within a single experiment. We next consider how action and language priming may interact.

### Priming Across Tasks

**FIGURE 5:**
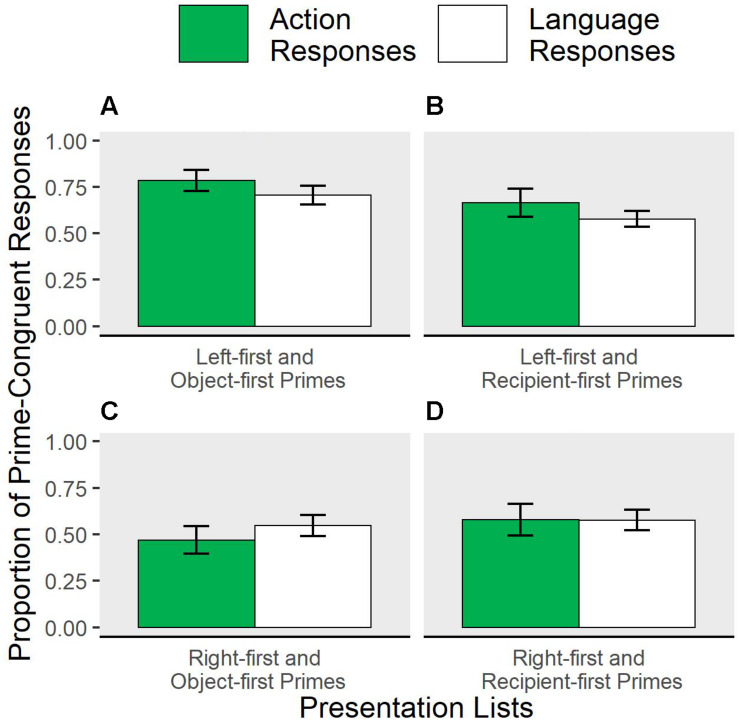
Influence of presentation list on proportion of primed responses. Action and language prime types varied between participants resulting in four combinations of primes (presentation lists). Proportion of prime-congruent action responses (green) and language responses (white) are shown grouped by presentation list. **(A)** Participants with Left-first and Object-first Primes. **(B)** Participants with Left-first and Recipient-first Primes. **(C)** Participants with Right-first and Object-first Primes. **(D)** Participants with Right-first and Recipient-first Primes.

Because the same participants completed both action and language trials in the same experiment, we can explore whether priming in one domain (action or language) affects rate of primed responses in the other. If action and language planning are related in some way, the effectiveness of plan reuse in one domain may impact behavior in the other. In our study, prime direction was manipulated between subjects, and combinations of priming direction in the two domains was counterbalanced across subjects via four different lists: left-first action prime + object-first language prime, left-first + recipient-first, right-first + object-first, and right-first + recipient-first primes. The patterns of priming in each list are shown in Figure 5. In exploratory analyses, we aimed to test whether the effectiveness of a prime in one domain was modulated by the direction of the prime in the other domain.

Responses in target trials (1 = prime-congruent, 0 = incongruent) were fit to domain (language vs. action), language prime (object-first vs. recipient-first), action prime (left-first vs. right-first), trial order, and random effects. An interaction between action and language prime conditions would suggest some form of influence of conditions in one task domain on the other task domain.

In this fully interactive model, the effect of prime was reliable across domains and prime levels (*z* = 4.56, *p* < 0.05). This result was expected, because primes had reliable effects in each domain when analyzed separately above. Further analyses show some evidence for priming effects to vary across the different combinations of action and language primes that participants experienced in the four presentation lists. Across all target responses, the likelihood of being primed was significantly higher if action primes were left-first instead of right-first, controlling for target domain (*z* = −2.79, *p* < 0.05). The size of the influence of action plans (domain^∗^action prime) depended on domain (a greater effect for action target responses; *z* = 1.97, *p* < 0.05). These effects are clarified by considering underlying main effects within individual presentation lists. Two significant main effects suggest that the source of the cross-domain modulation of priming is a mutual facilitation between left-first and object-first priming conditions. First, prime-congruent responses in the action task were more frequent for left-first primes than right-first in the presentation lists that also included object-first primes (filled bars of Figure 5A vs. 5C, *z* = −1.99, *p* < 0.05); this left-right priming difference was not obtained in the lists containing recipient-first language primes (filled bars in Figure 5B vs. 5D). Second, object-first language primes paired with left-first action primes were more effective in eliciting object-first picture descriptions compared to when object-first primes were paired with right-first action primes (white bars, Figure 5A vs. 5C, *z* = −2.9, *p* < 0.05). No other main effects were reliable.

While these effects emerged from exploratory analyses, they may suggest some cross-talk between the two tasks, potentially consistent with a domain general planning system.

## Discussion

We sought to develop parallel studies of two phenomena that have not previously been studied together, hysteresis in action and syntactic priming in language production. Both of these phenomena are well-established in their own fields, and we investigated whether an experimental design amenable to both action and language could elicit these effects in both domains in the same participants and within the same experiment. Consistent with previous research, we found that target responses for action and language tasks were both influenced by the structure of the immediately preceding behaviors that were carried out in response to the prime trials. Exploratory analyses suggested that left-first action primes and object-first language primes elicited the most priming when they were paired with each other in an experiment list.

This investigation of parallel Plan Reuse effects in each domain is necessarily preliminary, and any demonstration of parallel behaviors does not guarantee that the origin of the parallel effects is a single domain general system. Nonetheless, the attempt to put action and language tasks on the same footing, and the finding of comparable plan reuse effects, are interesting in several respects. In the next sections, we consider how researchers in action and language domains have interpreted plan reuse effects like the ones we have investigated as well as future applications of the methods we have introduced.

### Theoretical Accounts of Plan Reuse

Our study shows that plan reuse phenomena in both action and language can have similar behavioral profiles. As we noted, however, the hypothesized underlying mechanisms in each field are not necessarily aligned. Here, we discuss some key theoretical differences that may prove challenging for more fully integrating the two fields, which may present challenges for viewing language production planning as emergent from more general action planning systems. We will consider how our parallel action-language method could have a role in investigating these theoretical approaches.

In action research, hysteresis has often been viewed through a dynamical systems lens, in which repetition of past actions owes to task-specific attractor states, with little or no contribution of cognitive computation (e.g., Kelso et al., 1994). An alternative view is that hysteresis may emerge from a confluence of biomechanical and cognitive considerations, including computational efficiency gained from reusing a recent plan (Meulenbroek et al., 1993; Rosenbaum et al., 2012; Valyear et al., 2019). The computational efficiency approach is supported by both neural and behavioral data. For example, response times to initiate movements are reduced when actions are repeated (Valyear and Frey, 2014; Valyear et al., 2019), and there is reduced neural activity in areas involved in action planning (e.g., intraparietal and superior parietal cortex) when actions are repeated (Valyear and Frey, 2015). Moreover, the cognitive processes involved in generating a new motor plan may interfere with serial recall position effects, again suggesting a deeper relationship between cognition and planning for physical action (Weigelt et al., 2009).

There are potentially biomechanical effects on serial order in language production, such as preferences for ordering shorter words before longer ones (McDonald et al., 1993), but in contrast to the action hysteresis accounts described above, the reuse of syntactic structures in language are viewed as owing to cognitive efficiency biases, not to biomechanical factors. There are two reasons behind this cognitive emphasis. First, syntactic priming effects are thought to arise in an early stage of language production planning, before biomechanical factors come into play (McDonald et al., 1993). Second, syntactic priming effects don’t require overt production of a prime; several studies have found reliable syntactic priming effects both when producers overtly produced a prime sentence before a target and also when producers merely listened to someone else producing the prime sentence (Chang et al., 2000; Bock et al., 2007; Mahowald et al., 2016). This evidence of priming without overt action means that syntactic priming effects cannot be attributed to the physical state of a system following an overt action. Researchers argue instead for a cognitive origin of the priming effects, that both the cognitive processes involved in interpreting language input and the processes that plan for upcoming productions can bias a speaker to adopt the same syntactic structure that was encountered or produced earlier.

These different conceptions of plan reuse in action and language present some barriers to accounts of domain generality and emergence of language planning from action planning processes, but they also offer opportunities for future research. One possibility is to consider the role of learning in the implicit decision making that may govern plan reuse phenomena in both action and language. Bock and Griffin (2000) and Chang et al. (2000) have used both empirical results and computational simulations to argue that structural priming in language production (i.e., plan reuse) reflect implicit learning. Similarly, studies of implicit decision making and habits in other action domains, such as whether an animal goes down the left or right branch of a maze, have assumed a learning component in decision making processes for actions (e.g., Mattar and Daw, 2018; Piray and Daw, 2019). We expect that a greater attention to implicit decision making in language production and other non-linguistic choice tasks will prove important to pursuing potential links between language and non-linguistic action planning.

Another step to bridging the theoretical divide could be to adapt our paradigm to investigate whether both action and language behaviors can be primed via perception of a prime stimulus. Rather than reading language prime sentences aloud as in the current study, participants could listen to the prime sentences presented via a computer or live confederate in a joint action task (Branigan et al., 2000), and rather than producing actions on prime trials, participants could watch the prime action being completed via video or live confederate. Some secondary task would likely be required to insure that participants paid attention to the primes (Bock et al., 2007), and the action trials would need to avoid priming sequences of eye movements when viewing the primed actions. Reliable priming from perceptual primes in a study of this sort would suggest that overt action is not strictly necessary for plan reuse. Such results would instead argue for a more cognitive approach to hysteresis in action, better aligned with the approach in language. Indeed, there are arguments in action research that the same processes that monitor an agent’s own action also can be engaged to interpret and align with another’s actions (Wolpert et al., 2003), suggesting a computational basis for action priming via action perception. Alternatively, a finding of perceptual priming only in language, not in action, would argue against domain general accounts. Any differences in strength of priming in the prime-production and prime-perception conditions in each domain could add further information concerning the degree to which plan reuse in action and language appear similar.

### Interactions Between Action and Language Tasks

Potential cross-talk between interleaved action and language tasks also merits further investigation. Our exploratory analysis showed that the left-first action condition led to more effective object-first priming in the language task relative to the right-first action condition. Further, only in the context of object-first language primes, the left-first primes were more effective than right-first primes in the action task. Given that left-first touches appear to be the preferred pattern in our action task and object-first sentences are sometimes the dominant response in language production studies (Bock and Irwin, 1980; Bock and Loebell, 1990), it is possible that priming these preferred forms together resulted in less effortful planning overall, perhaps as a consequence of placing fewer demands on cognitive mechanisms common to both tasks (akin to the argument made by Weigelt et al., 2009). Although it is difficult to precisely determine the locus and robustness of these effects in the present study, the results are promising enough to warrant a closer and more systematic look at the parallels between planning for action and for language production with an eye toward understanding whether and to what extent they draw from similar cognitive substrates. Other types of cross-talk between language and action tasks are also potentially interesting but with uncertain interpretations. Some researchers have found that certain non-linguistic perception or action tasks themselves prime certain sentence structures in language production (Kaiser, 2012; Scheepers and Sturt, 2014; Van de Cavey and Hartsuiker, 2016). One interpretation of these data is that they reflect domain general sequencing mechanisms (Van de Cavey and Hartsuiker, 2016), but an alternative is that these effects reflect a domain-general representation of events, so that priming of certain event representations have potential to affect both action planning and verbal descriptions of events (Kaiser, 2012; see also Ziegler et al., 2018; Gruberg et al., 2019). Another possibility is that shared planning is driven by shared (external) organization. For example, left-first action plans might guide visual scanning of pictured events with the agent, object and recipient appearing left-to-right (8 out of 12 of our language target pictures) and subsequently elicit more object-first language planning. These accounts are not mutually exclusive—there may exist both domain general representations of events and a domain general sequencing system, and future research should be directed at addressing the alternative theorizing here.

## Conclusion

Steedman (2002) argued that ideas relating language to action have been implicit in theorizing in both fields for over a century, and Lashley’s (1951) explicit linkages between action and language production have shaped thinking for decades. These ideas hold promise for conceiving of key properties of language use as emergent from other systems. However, there has been relatively little contact between the fields of action and language production, and different underlying assumptions of the mechanisms that give rise to effects such as plan reuse. Our own study, with its similarly structured language and action priming tasks, encourages discussion across the two domains and offers some steps toward further investigation of language and action relationships.

## Data Availability Statement

The datasets generated for this study are available on request to the corresponding author.

## Ethics Statement

The studies involving human participants were reviewed and approved by Social Sciences IRB, University of Wisconsin-Madison. The patients/participants provided their written informed consent to participate in this study.

## Author Contributions

MM and DW designed the study. MK and FB developed the stimuli with input from MM. MK and FB directed participant testing. MK conducted the data analyses. All authors wrote the manuscript.

## Conflict of Interest

The authors declare that the research was conducted in the absence of any commercial or financial relationships that could be construed as a potential conflict of interest.
