# Supplementary material for: Is Language Production Planning Emergent From Action Planning? A Preliminary Investigation
Source: Front Psychol. 2020 Jun 5;11:1193. doi: 10.3389/fpsyg.2020.01193 (PMC7290767; doi:10.3389/fpsyg.2020.01193)
Supplement: Supplementary file 1 [file Data_Sheet_1.docx]

Supplementary Material

# Action target images


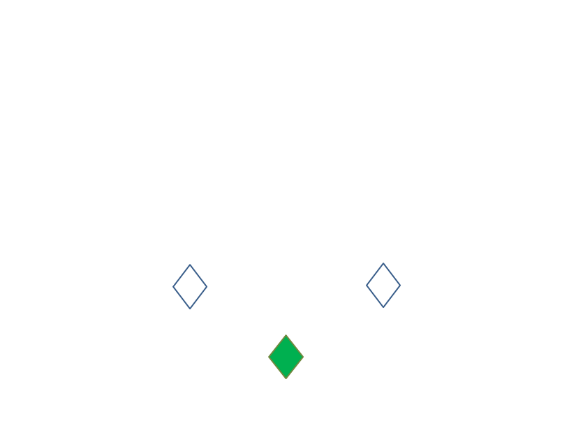

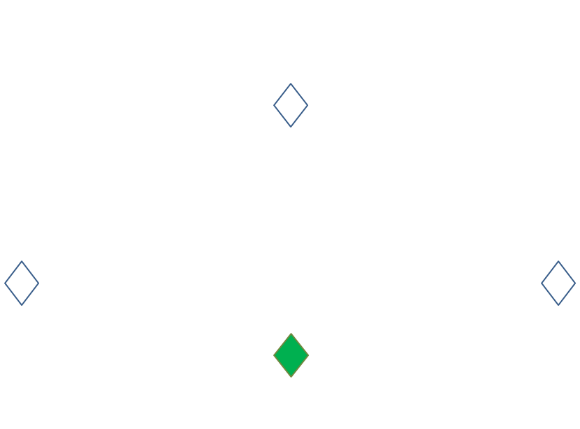

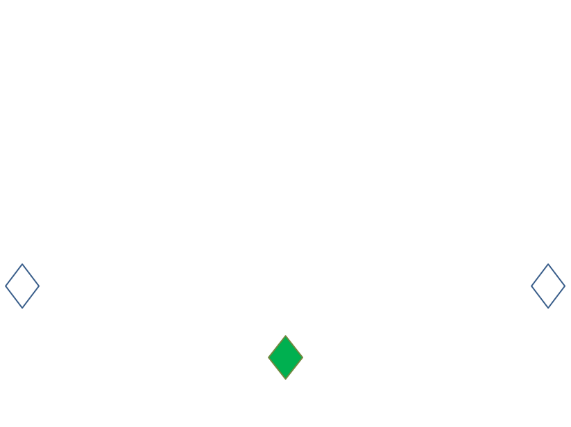

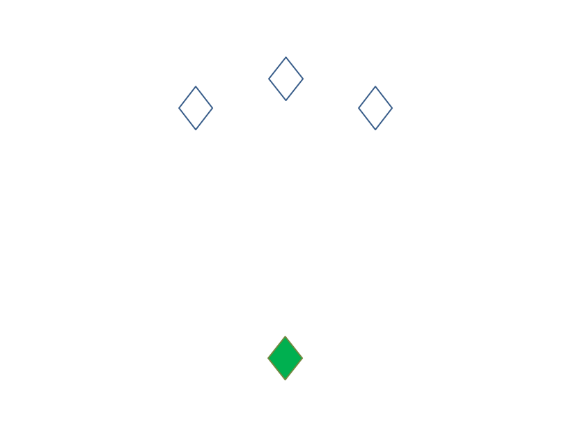

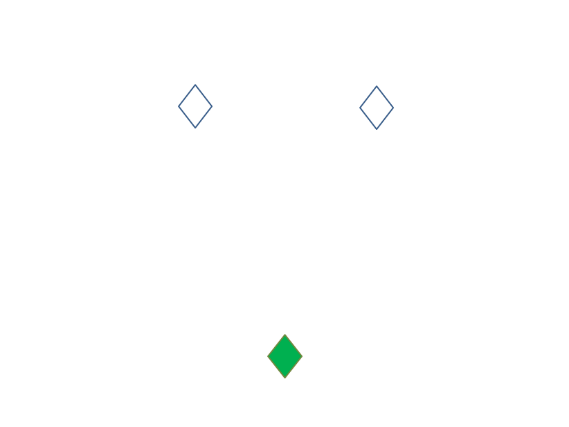

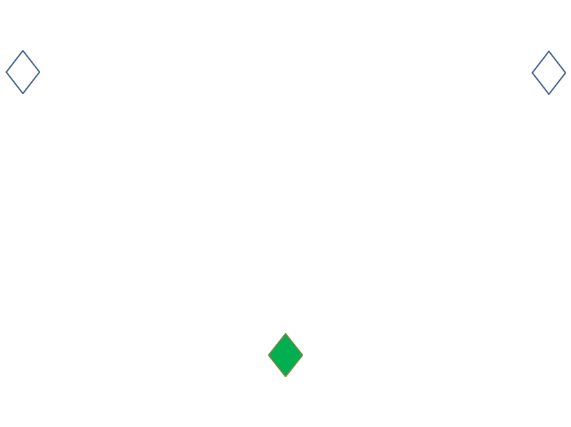


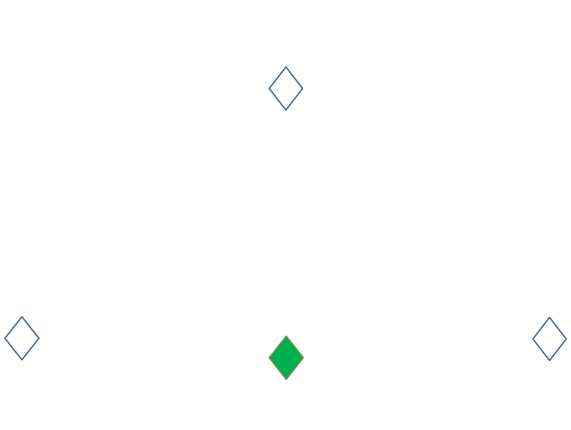

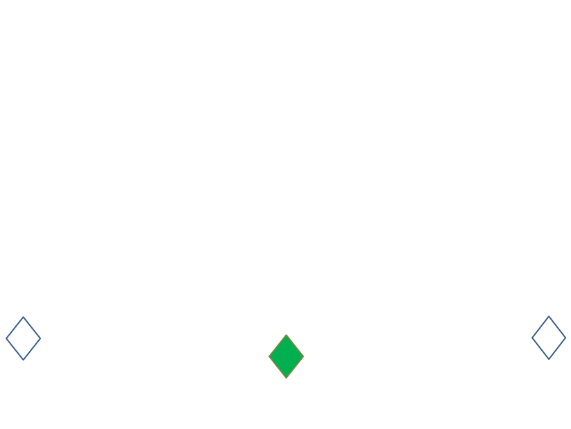

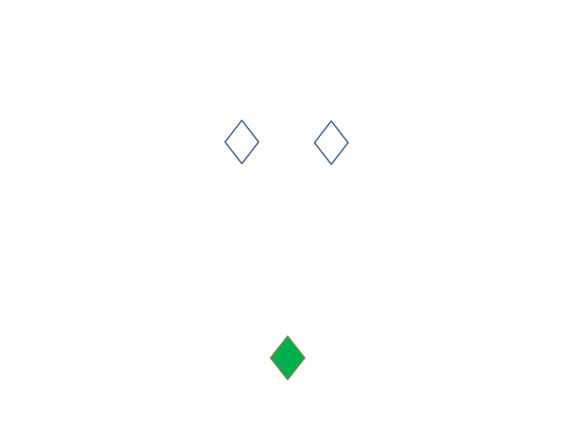

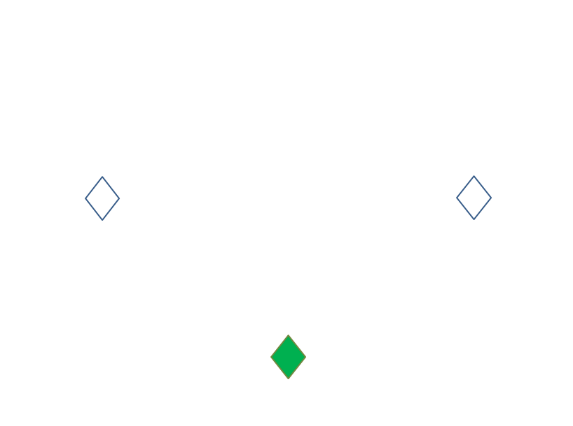

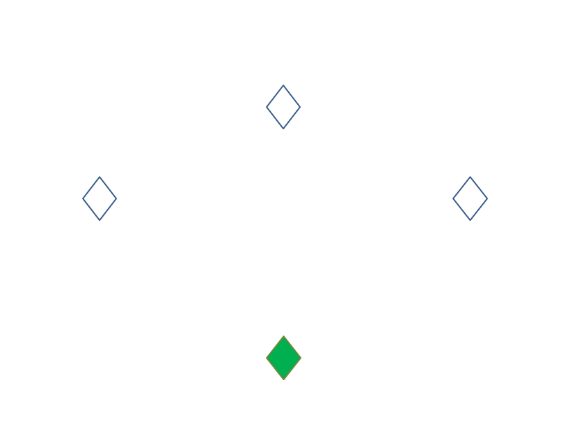

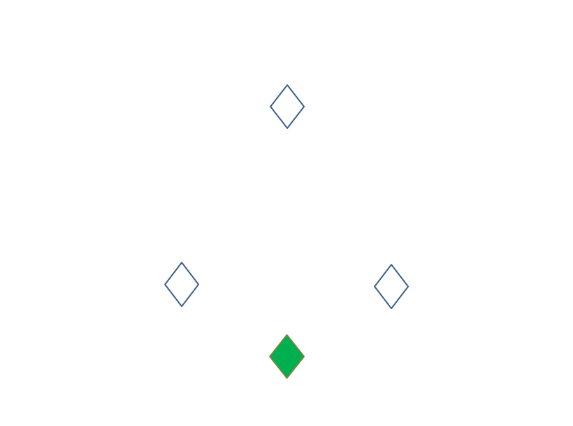


# Language target images


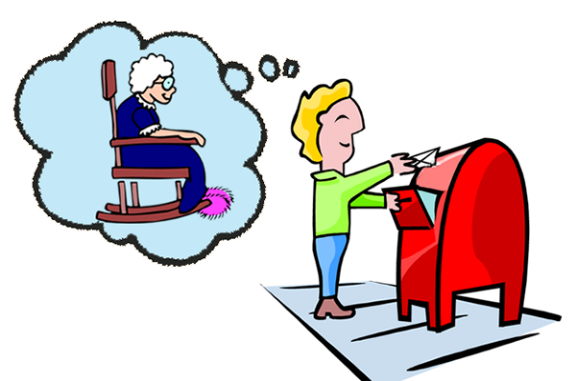

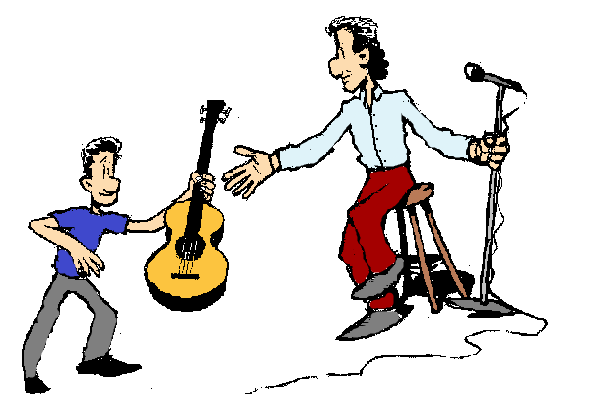

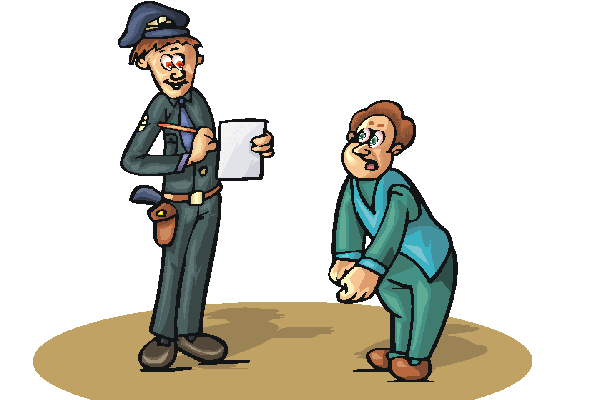

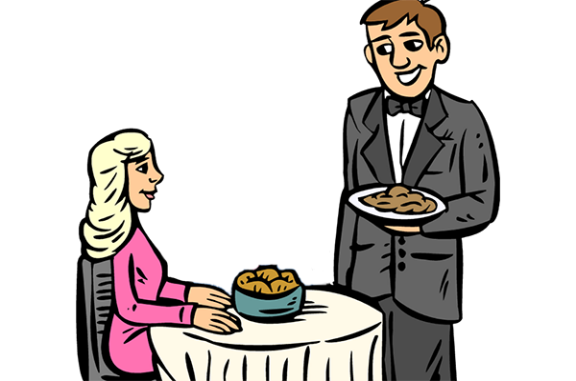

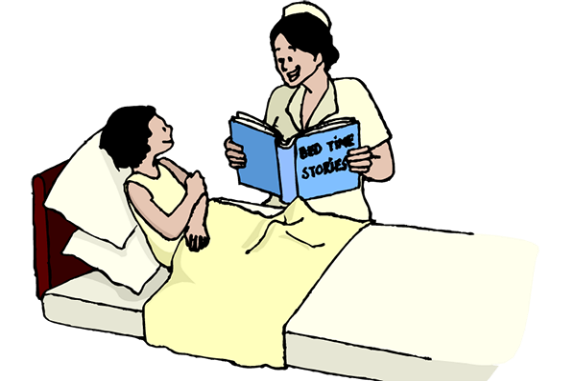

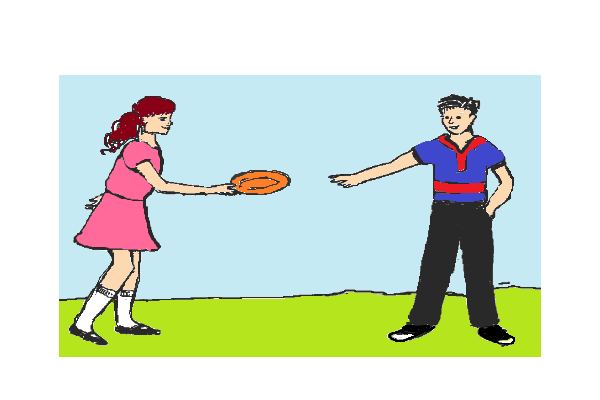

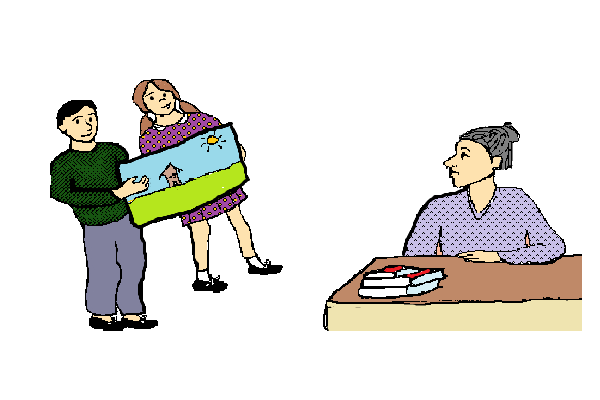

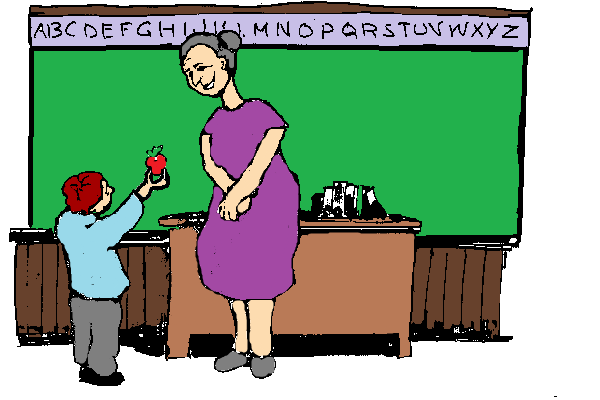

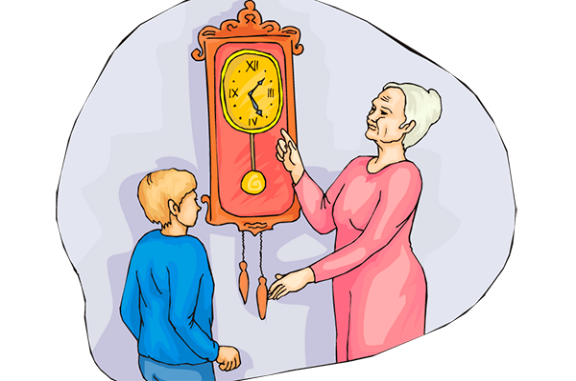

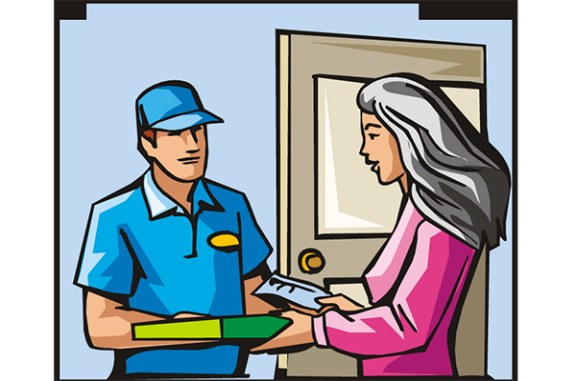

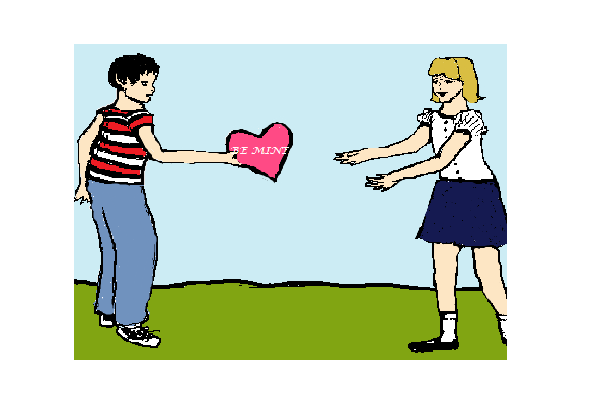

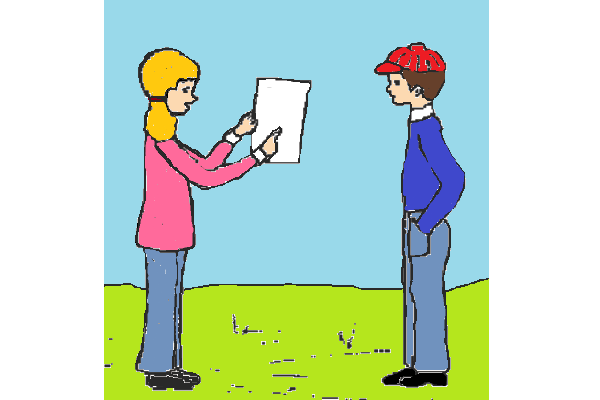


# Language primes

## For- alternations in Recipient-first form

These appeared first in prime pairs for language items.

The babysitter fixed the child a sandwich. (a sandwich for the child.)

The grandma knit the girl a scarf.

The soprano sang the judge a song.

The man wrote his girlfriend a play.

The scientist found the museum owner a new bone.

The girl saved her brother a cupcake.

The bartender poured his friend a shot.

The lifeguard built the toddler a sand-castle.

The new assistant bought the employee a coffee.

The student drew his teacher a picture.

The artist painted his wife a painting.

The mom ordered her son a sweater.

## To- alternations in Recipient-first form

The cheerleader gave her friend a seat.

The chef passed the butler some scrambled eggs.

The grandmother sent her granddaughter a quilt.

A gardener sold the woman some flowers.

The teenager handed his brother a model ship.

The maid brought the hotel guest a towel.

The father threw his six-year-old a baseball.

An artist showed the police captain a sketch.

A soldier offered his pal a cigarette.

The teacher gave the preschooler a sticker.

The guard tossed the prison warden the keys.

A seamstress sent the bride-to-be a wedding dress.
